# Gender and the double burden of economic and social disadvantages on healthy eating: cross-sectional study of older adults in the EPIC-Norfolk cohort

**DOI:** 10.1186/s12889-015-1895-y

**Published:** 2015-07-22

**Authors:** Annalijn I. Conklin, Nita G. Forouhi, Paul Surtees, Nicholas J. Wareham, Pablo Monsivais

**Affiliations:** UK Clinical Research Collaboration Centre for Diet and Activity Research (CEDAR), MRC Epidemiology Unit, University of Cambridge School of Clinical Medicine, Cambridge Biomedical Campus, Cambridge, UK; Medical Research Council Epidemiology Unit, University of Cambridge School of Clinical Medicine, Cambridge, UK; Strangeways Research Laboratory and Department of Public Health and Primary Care, University of Cambridge, Cambridge, UK

**Keywords:** Gender, Fruit and vegetable intake, Social relationships, Economic determinants, Financial hardships, Deprivation amplification, Aging, EPIC cohort

## Abstract

**Background:**

Multiple economic factors and social relationships determine dietary behaviours, but the inter-relations between determinants is unknown. Whether women and men differ in the vulnerability to, and impact of, combined disadvantages is also unclear. We examined associations between diverse combinations of economic resources and social relationships, and healthy eating in British older women and men.

**Methods:**

Our sample comprised 9,580 over-50s (47 % of over-50 respondents) in the EPIC-Norfolk cohort study. We examined six economic factors (education, social class, home-ownership, money for needs, frequency of insufficient money for food/clothing, paying bills) and three social relationships (marital status, living arrangement and friend contact), independently and in combination, in relation to fruit variety and vegetable variety. We analysed gender-specific associations using multivariable linear regression with interaction terms.

**Results:**

Lower social class, lower education, and difficulty paying bills were associated with lower fruit and vegetable variety in both genders, independent of social relationships. All social relationships were independently associated with fruit variety in men and with vegetable variety in both genders. Substantially lower variety was found for all combinations of low economic resources and lack of social relationship than for either measure alone, with men faring worse in the majority of combined disadvantages. For example, the difference in vegetable variety for men reporting low social class and non-married was much greater (β -4.1, [-4.8, -3.4]), than the independent association of low social class (β -1.5, [-1.8,–1.2]), or non-married (β -1.8, [-2.3,–1.3]). Variety was also lower among men with high economic resources but non-married or lone-living.

**Conclusion:**

A double burden of low economic resources and lack of social relationships suggested they are unique joint determinants, particularly in older men, and that public health efforts to improve healthy eating would offer most benefit to older adults with intersecting economic and social disadvantages.

**Electronic supplementary material:**

The online version of this article (doi:10.1186/s12889-015-1895-y) contains supplementary material, which is available to authorized users.

## Background

As older adults comprise more of the population, there is increasing importance to promote healthy eating so as to prevent and reduce chronic conditions and thereby support healthy ageing. Consumption of fruits and vegetables (FV) is critical to healthy eating as inadequate intakes contribute to many chronic diseases and nearly 5 % of excess deaths globally [1]. Importantly, both quantity and variety of FV intake are independently associated with reduced risk of diabetes and some cancers [2, 3]. Eating a greater variety of FV is especially valuable for older adults who require nutritionally dense diets with lower energy intakes [4]. However, older age correlates with poorer economic resources and fewer social relationships, and both are linked to lower FV consumption [5, 6]. In particular, variety of FV intakes is lower among non-married, lone-living and socially isolated older adults, particularly men [7]. Similarly, FV variety was lower among older adults with lower education, lower social class, and greater financial hardships [8].

Current research on the determinants of diet in older adults is still limited and there has been no study of the inter-play of multiple economic circumstances with different social relationships [9]. A small study of US older adults found greater financial stress correlated with adverse dietary effects, but these adverse effects were buffered by companionship [10]. Others show more generally that having a confidant relationship can mitigate older people’s subsequent response to life stresses [11]. As suggested by Intersectionality Theory [12, 13], it is therefore possible that social relationships constitute an asset (resource) generating economic resources and vice versa, and thus absence of both creates a double burden of intersecting disadvantages with a unique impact on healthful dietary behaviours. Most importantly, the independent effects of single factors (e.g. social class or marital status) do not fully describe the influence of diverse combinations of two factors (e.g. low social class and married, or high social class and non-married [13–15]. Thus an important gap in public health knowledge remains despite common understanding that individuals are highly differentiated in the combination of economic and social categories they occupy.

Furthermore, women and men differ in their exposure to low economic resources or fewer social relationships, and in their vulnerability to poor health outcomes [16, 17]. Older women are typically more disadvantaged economically and report greater financial hardships than men [18], which in turn constrains women’s food procurement and preparation [19]. Older men living or eating alone are more at risk of poor diets than women [20]; a risk that can be compounded by material deprivation [21]. Older single and married men are more likely to be dependent on others for food-related decision-making than are older women [22]. Thus, it remains an empirical question to determine if one gender is overall more vulnerable to less healthy eating from both poor economic and social resources, or if women’s and men’s vulnerability varies by which combination is specified. How inter-relations between diverse economic and social resources influence healthy eating therefore deserves further exploration in older women and men.

This cross-sectional study examined the inter-relations of multiple economic resources and social relationships on variety of FV consumption, as a measure of healthy eating, in British older adults. We hypothesized that lack of social relationships would amplify associations between poor economic resources and lower variety. We also expected that women and men would differ in the specific combination of economic resources and social relationships associated most strongly with FV variety.

## Methods

### Study population

We used data collected in the population-based EPIC-Norfolk cohort which recruited 25,639 men and women aged 39 to 79 (99.7 % white) from age-sex registers of general practices, who attended a first health check at entry (1993–97) (33 % of those invited) [23]. As we are interested in life circumstances of adults near the end of working life and beyond, our eligible sample comprised 20,274 over-50s who had similar socio-demographics and health behaviours as the full cohort at entry [7, 24]. Information on education and social class were collected at entry; the remaining exposure data on financial hardships and social relationships were self-reported in a postal questionnaire by 50–71 % of cohort participants (1996–2000). Responses to individual questions from over-50s ranged between 10,352 and 17,998 (51–89 % response rate). Diet data were collected from 12,292 (48 %) cohort participants using a Food Frequency Questionnaire (FFQ) during a second health check (1998–2002). The EPIC-Norfolk FFQ was previously validated against a 16-d weighed food record [25], and nutrient biomarkers [26]. Outcome data from over-50s (*n* = 9933, 49 %) was restricted to FFQ respondents for whom plausible total daily energy (kcal) could be derived, as defined by top and bottom 0.5 percentile of energy intake relative to basal metabolic rate [27]. Thus, our available sample included over-50s who responded to questions on economic and social resources, had covariates and follow-up dietary data (*n* = 9580). All volunteers gave written informed consent and the study was approved by the Norwich district ethics committee.

### Measures

#### Financial hardship and conventional socioeconomic status

Economic resources were examined using three conventional indicators of socioeconomic status (education, social class and home-ownership) and three novel measures of financial hardship (money for needs, frequency of not enough money for food/clothing and difficulty paying bills), as previously studied [24]. Social resources were assessed using three types of structural social relationships with known associations with healthful eating in our sample: namely, marital status, living arrangement and friend contact [7]. As most measures comprised several categories, we dichotomised at mid-point to construct new variables of four categories to examine different combinations of economic resources and social relationships (see Table 1). Thus, combination variables were constructed by defining, for example, high education and married/high education and non-married/low education and married/low education and non-married; the reference category was high economic and social resources (e.g. high education and married).

**Table 1:** Overview of variables constructed for data analysis

Exposures of Interest	Dichotomisation approach	
	*‘High’ economic resource*	*‘Low’ economic resource*
Social class	Professional, managerial & technical, skilled non-manual	Skilled manual, partly skilled, unskilled
Education	Degree, A-level	O-level, no qualification
Home-ownership	Owner-occupier	Renter-private, renter-public
Money for needs	More than enough	Just enough, less than enough
Frequency of insufficient money for food/clothing	Never, seldom	Sometimes, often, always
Paying bills (level of difficulty)	None, slight, a little	Some, great, very great
	*‘Present’ social relationship*	*‘Absent’ social relationship*
Marital status	Married, living as married (“married”)	Single, widowed, divorced, separated (“non-married”)
Living arrangement	Co-living	Lone-living
Frequency of friend contact	Daily, weekly, several times a month (“frequent”)	About once a month, less than once a month, never/hardly ever (“infrequent”)

Social class (*n* = 9407) had 6 categories based on the UK Registrar General’s classification of occupations, with the top three categories of non-manual occupations used to define high social class (versus low social class comprised of three categories of manual occupations). Education’s (*n* = 9574) top two categories (university/college/equivalent degree and A-level (education to age 18 years) were grouped to define high education compared to low education based on O-level (to age 16 years) and no qualification. Four categories of accommodation (*n* = 8829) were used as a proxy for older adults’ wealth by distinguishing ‘home-owners’ from those who rented public and private (furnished and unfurnished) accommodations. Having more than enough money for needs (top category) was distinguished from just enough and less than enough responses (bottom two categories) (*n* = 8747). Frequency of having not enough money to afford adequate food/clothing (hereafter, ‘frequency of insufficient money for food/clothing’) included response categories never and seldom (versus sometimes, often and always categories) (*n* = 8753). The question on level of difficulty in paying bills (*n* = 8762) comprised three top response categories of none, slight, and a little (versus some, great, and very great response categories). The presence of each type of financial hardship was considered to reflect participants’ low economic resources.

#### Social relationships

Marital status (*n* = 6257) had six response categories which were dichotomised as ‘married’ (married and living as married) and ‘non-married’ (single, widowed, divorced and separated). Living arrangement (*n* = 8816) was collected as a binary measure, with co-living as the reference. Finally, a question about frequency of contact with a friend (*n* = 8442) had seven response categories, with frequent friend contact defined by responses daily, weekly, and several times a month (versus ‘infrequent’ by about once a month, less than once a month and never/hardly ever). Non-married, lone-living and infrequent friend contact, were considered to reflect participants’ absence (lack) of a given social relationship.

#### Variety of FV consumption

Dietary data from the semi-quantitative FFQ were used to calculate scores for variety of intake fruits or vegetables. Participants reported their average consumption of a pre-specified number of fruit (*n* = 11) and vegetable (*n* = 26) products over the last year, with nine standard response categories (never/less than once/month to ≥ six/d) [28]. Average daily consumption of each item (g/d) was calculated from self-reported frequencies using an established method [27]. Using data on daily consumption, we summed the total number of unique products to derive a score for fruit variety (0–11) and vegetable variety (0–26), irrespective of total quantity (>0 g/d). The score (no. items/month) corresponded to average frequency intakes of at least 1–3 times per month, which reflects the minimum two weeks needed to exhaust the variety of one’s food repertoire [29]. It followed a similar approach previously demonstrated for disease risk reduction in this cohort [2, 3], and thus serves as an indicator for healthfulness of the diet. Others have demonstrated the reproducibility and validity of variety scores for nutritional adequacy and diet quality in older adults [29, 30].

#### Socio-demographic variables

Concurrent socio-demographic variables included: self-rated general health status (excellent/good/fair/poor), smoking status (current/former/non), physical activity and energy expenditure (PAEE score range: 0–1428, kilojoules per day), and total alcohol intake (units/week). Age (continuous) and gender (binary) were assessed at entry but are generally time-invariant.

### Data analysis

Descriptive statistics characterised our sample across categories of single economic (social class, education, paying bills) and social (marital status, living arrangement, friend contact) resources and economic-marital status combinations. Three sets of multivariable linear regression analyses examined gender-specific associations of economic, social and combined exposures with variety scores. First, each economic or social variable was entered separately into models adjusting for age and total energy intake which included an interaction term between sex and exposure variables (significant gender differences set to *p*<0.10). Second, analyses of economic exposures were additionally adjusted for social exposures, and vice versa. Third, analyses explored how economic resources varied in associations with variety scores when a social relationship was considered. For simplicity, main results are given for three economic measures examined (education, social class and bill payment) and supplementary files provided for the remainder. Results are presented as regression coefficients and 95 % confidence intervals (CI95). Given our data were actual observations, we followed recommendations of not adjusting for multiple comparisons because doing so would increase the risk of type II error for those associations that are not null [31]. All analyses were performed using Stata 12.1.

## Results

Our sample’s age averaged 62 years, with 55 % women. Most (83 %) rated their general health as good/excellent, 51 % were ever smokers, and over half had degree/A-level education. Notably, more of the men (62 %) than women (48 %) had higher education levels. Normally distributed scores for fruit variety and vegetable variety were higher in woman than men (7.7 and 16.5 versus 6.7 and 15.9, respectively). Few over-50s reported no average daily consumption of fruits (*n* = 55) or vegetables (*n* = 6) and thus scored zero.

Between 175 and 959 over-50s reported both low economic resources and absent social relationships, such as low education and non-married. As shown in Table 2, the sample’s characteristics differed between categories of economic resources and social relationships, or across categories of the economic-marital status combinations only with respect to gender, self-rated general health, and lifestyle factors. For example, a higher proportion of over-50s reporting moderate/poor general health were found in the categories of low economic resource and absent social relationships, with the highest proportions observed in the combined category of both low economic resource and non-married.

**Table 2:** Distribution of socio-demographic characteristics in over-50s in the EPIC-Norfolk study across single or combined economic and social resources

	Mean (SD) age	Women	Poor/moderate health	Ever smoker	Mean (SD) PAEE score	Mean (SD) alcohol intake (units/week)	Mean (SD) BMI (kg/m^2^)	Mean (SD) fruit variety score (0-11)	Mean (SD) vegetable variety score (0-26)
Social class (*n* = 9407)									
High (*n* = 5980)	62 (7)	56 %	14 %	49 %	49 (51)	8.6 (9.1)	27 (4)	7.5 (2.4)	16.7 (3.9)
Low (*n* = 3427)	62 (7)	54 %	21 %	55 %	57 (63)	5.5 (8.2)	27 (4)	6.9 (2.5)	15.5 (4.1)
Education (*n* = 9574)									
High (*n* = 5200)	62 (7)	49 %	14 %	51 %	57 (59)	8.0 (9.5)	27 (4)	7.5 (2.4)	16.8 (3.9)
Low (*n* = 4374)	63 (7)	63 %	20 %	52 %	46 (51)	5.4 (7.7)	27 (4)	7.0 (2.5)	15.6 (4.0)
Paying bills (*n* = 8762)									
No difficulty (*n* = 8038)	62 (7)	55 %	15 %	51 %	52 (56)	6.9 (8.9)	27 (4)	7.3 (2.4)	16.3 (4.0)
Difficulty (*n* = 724)	61 (8)	60 %	30 %	57 %	52 (52)	5.6 (9.0)	28 (5)	6.9 (2.6)	15.7 (4.3)
Marital status (6257)									
Married (*n* = 5040)	62 (7.0)	52 %	15 %	50.5 %	53 (53)	7.0 (8.7)	27 (4)	7.3 (2.4)	16.4 (3.9)
Non-married (*n* = 1217)	64 (8)	76 %	21 %	49.5 %	43 (45)	5.6 (8.8)	27 (5)	7.3 (2.5)	15.3 (4.4)
Living arrangement (*n* = 8816)									
Co-living (7243)	62 (7)	52 %	16 %	52 %	53 (56)	7.0 (8.9)	27 (4)	7.3 (2.4)	16.4 (3.9)
Lone-living (1573)	65 (7)	71 %	19 %	50 %	43 (54)	5.8 (8.7)	27 (4)	7.2 (2.5)	15.4 (4.3)
Friend contact (*n* = 8442)									
Frequent (*n* = 6972)	62 (7)	58 %	16 %	49.5 %	51 (57)	7.0 (9.0)	27 (4)	7.4 (2.4)	16.5 (3.9)
Infrequent (*n* = 1470)	62 (7)	45 %	19 %	57 %	53 (52)	6.4 (8.6)	27 (4)	6.8 (2.5)	15.6 (4.2)
Social class and marital status (*n* = 6,151)									
High class, married (*n* = 3,156)	62 (7)	51 %	13 %	49 %	50 (49)	7.9 (9.3)	27 (4)	7.5 (2.3)	16.9 (3.8)
High class, non-married (*n* = 764)	64 (8)	79 %	18 %	47 %	44 (41)	6.0 (9.0)	26 (4)	7.6 (2.3)	15.9 (4.3)
Low class, married (*n* = 1,825)	61 (7)	53 %	19 %	54 %	58 (59)	5.5 (7.4)	27 (4)	7.0 (2.5)	15.6 (3.9)
Low class, non-married (*n* = 406)	65 (7)	71 %	25 %	56 %	45 (54)	4.5 (7.8)	28 (5)	6.7 (2.7)	14.1 (4.3)
Education and marital status (*n* = 6,252)									
High education, married (*n* = 2,820)	61 (7)	44 %	13 %	50 %	58 (57)	8.2 (9.4)	27 (4)	7.4 (2.4)	16.9 (3.8)
High education, non-married (*n* = 642)	63 (8)	74 %	17 %	49 %	48 (48)	6.8 (9.9)	26 (5)	7.6 (2.4)	16.2 (4.1)
Low education, married (*n* = 2,216)	62 (7)	61 %	18 %	51 %	46 (47)	5.5 (7.4)	27 (4)	7.1 (2.4)	15.8 (3.9)
Low education, non-married (*n* = 574)	65 (7)	79 %	25 %	51 %	38 (42)	4.1 (7.0)	27 (5)	6.9 (2.7)	14.3 (4.5)
Paying bills and marital status (*n* = 5,839)									
No difficulty, married (*n* = 4,388)	62 (7)	52 %	14 %	50 %	53 (53)	7.1 (8.8)	27 (4)	7.3 (2.4)	16.5 (3.8)
No difficulty, non-married (*n* = 967)	64 (7)	75 %	17 %	49 %	44 (46)	5.8 (8.3)	27 (4)	7.4 (2.5)	15.4 (4.4)
Difficulty paying bills, married (*n* = 309)	61 (7)	48 %	26 %	58 %	57 (55)	6.0 (7.9)	28 (5)	7.0 (2.5)	16.0 (4.4)
Difficulty paying bills, non-married (*n* = 175)	62 (8)	80 %	40 %	57 %	41 (43)	4.8 (11.3)	28 (5)	6.9 (2.7)	15.2 (4.2)

Measurement time-points were: sex, age, education and occupational social class (1993-97); marital status, living arrangement, friend contact, money for needs, frequency of insufficient money for food/clothing, difficulty paying bills, physical activity and energy expenditure (PAEE) (1996-2000); self-rated general health, smoking status, and food and alcohol intakes (1998-2002)

### Independent associations of economic resources or social relationships with FV variety

We found low social class and low education were each associated with lower variety of intake of fruits or vegetables in both women and men, independent of their social relationships (Table 3, Model 2). Associations appeared stronger in men for social class, but in women for education. In terms of financial hardship, difficulty paying bills was independently associated with lower fruit variety in women only. Independent of multiple economic resources, non-married, lone-living and infrequent friend contact were each associated with lower fruit variety in men (Table 4, Model 2). By contrast, only friend contact was independently associated with fruit variety in women. In both women and men, there was an independent association between each social relationship and vegetable variety.

**Table 3:** Associations of economic resources with variety of fruit or vegetable intakes in older adults in the EPIC-Norfolk study

	Fruit Variety				Vegetable Variety			
	Women		Men		Women		Men	
	*Model 1*	*Model 2*	*Model 1*	*Model 2*	*Model 1*	*Model 2*	*Model 1*	*Model 2*
Social class								
High	reference	reference	reference	reference	reference	reference	reference	reference
Low	−0.43^a^	−0.50	−0.71^a^	−0.57	−1.15^a^	−1.30	−1.59^a^	−1.49
	(-0.56, -0.30)	(-0.67, -0.33)	(-0.86, -0.56)	(-0.76, -0.38)	(-1.37, -0.93)	(-1.58, -1.02)	(-1.84, -1.35)	(-1.82, -1.17)
	***	***	***	***	***	***	***	***
Education								
High	reference	reference	reference	reference	reference	reference	reference	reference
Low	−0.61	−0.61^a^	−0.52	−0.36^a^	−1.39	−1.61^a^	−1.17	−1.05^a^
	(-0.73, -0.48)	(-0.77, -0.45)	(-0.67, -0.38)	(-0.55, -0.17)	(-1.60, -1.18)	(-1.87, -1.34)	(-1.41, -0.93)	(-1.37, -0.73)
	***	***	***	***	***	***	***	***
Paying bills								
No difficulty	reference	reference	reference	reference	reference	reference	reference	reference
Difficulty	−0.54	−0.50^a^	−0.43	−0.11^a^	−0.72	−0.42	−0.82	−0.56
	(-0.77, -0.31)	(-0.79, -0.21)	(-0.71, -0.15)	(-0.46, 0.24)	(-1.10, -0.33)	(-0.91, 0.07)	(-1.28, -0.35)	(-1.15, 0.04)
	***	**	**		***		***	

Gender-specific beta coefficients (CI95) obtained by linear regression models using an interaction term and adjusting for age and energy intake (Model 1), and then for social relationships (marital status, living arrangement and frequency of friend contact) (Model 2). Numbers were: social class (Model 1: 9,407; Model 2: 5,522); education (Model 1: 9,574; Model 2: 5,608); paying bills (Model 1: 8,762; Model 2: 5,582). *p<0.05, **p<0.01, ***p<0.001; ^a^Significant gender difference (p-interaction<0.10)

**Table 4:** Associations of social relationships with variety of fruit or vegetable intakes in older adults in the EPIC-Norfolk study

	Fruit Variety				Vegetable Variety			
	Women		Men		Women		Men	
	*Model 1*	*Model 2*	*Model 1*	*Model 2*	*Model 1*	*Model 2*	*Model 1*	*Model 2*
Marital Status								
Married	reference	reference	reference	reference	reference	reference	reference	reference
Non-married	−0.08^a^	−0.09^a^	−0.62^a^	−0.37^a^	−0.76^a^	−0.75^a^	−2.07^a^	−1.79^a^
	(-0.25, 0.10)	(-0.28, 0.10)	(-0.90, -0.34)	(-0.67, -0.07)	(-1.06, -0.46)	(-1.06, -0.44)	(-2.55, -1.60)	(-2.29, -1.30)
			***	*	***	***	***	***
Living arrangement								
Co-living	reference	reference	reference	reference	reference	reference	reference	reference
Lone-living	−0.16	−0.16	−0.35	−0.24	−0.57^a^	−0.60^a^	−1.51^a^	−1.32^a^
	(-0.32, -0.00)	(-0.33, 0.00)	(-0.58, -0.12)	(-0.47, -0.00)	(-0.84, -0.31)	(-0.87, -0.33)	(-1.89, -1.12)	(-1.71, -0.94)
	*		**	*	***	***	***	***
Friend contact								
Frequent	reference	reference	reference	reference	reference	reference	reference	reference
Infrequent	−0.54	−0.42	−0.52	−0.49	−0.81	−0.53	−0.83	−0.68
	(-0.73, -0.34)	(-0.61, -0.22)	(-0.70, -0.34)	(-0.67, -0.30)	(-1.13, -0.49)	(-0.85, -0.20)	(-1.14, -0.53)	(-0.98, -0.38)
	***	***	***	***	***	**	***	***

Gender-specific beta coefficients (CI95) obtained by linear regression models using an interaction term and adjusting for age and energy intake (Model 1), and then for economic resources (social class, education, home-ownership, money for needs, frequency of insufficient money for food/clothing, paying bills) (Model 2). Numbers were: marital status (Model 1: 6,257; Model 2: 5,628); living arrangement (Model 1: 8,816; Model 2: 8,414); and frequency of friend contact (Model 1: 8,442; Model 2: 8,086). *p<0.05, **p<0.01, ***p<0.001; ^a^Significant gender difference (p-interaction<0.10)

Overall and independent associations of home-ownership, money for needs and frequency of insufficient money for food/clothing, with variety scores are given online (see Additional file 1: Table S1).

### Association of inter-relations between multiple economic resources and social relationships with FV variety

**Fig. 1:**
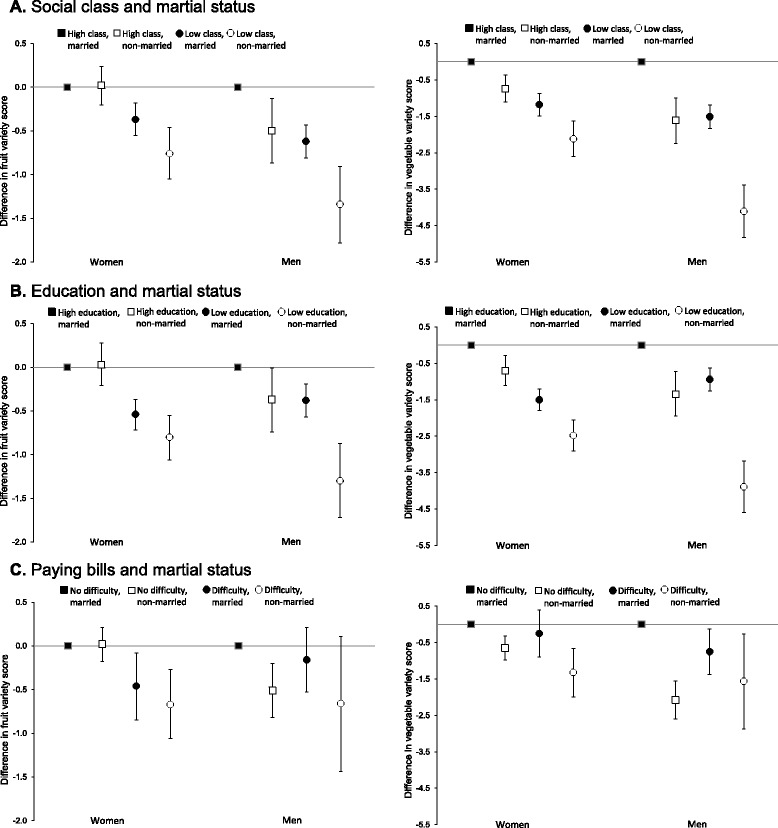
Association of social class, education and difficulty paying bills with variety of fruit or vegetable intake in older women and men by marital status. Left, fruit variety; Right, vegetable variety. Panel **a** is social class and marital status (*n* = 6151); **b**, education and marital status (*n* = 6252); **c**, difficulty paying bills and marital status (*n* = 5839)

The combination of marital status with social class, education or paying bills appeared to alter the independent associations previously observed between each economic factor and variety of fruit or vegetable intake (Fig. 1). Figure 1 illustrates how results for each economic resource variable, combined with married and non-married categories, revealed important heterogeneity for women and men. Overall, compared to reference groups, fruit variety was much lower in women and men reporting low economic resources and non-married status than in those reporting only low social class, low education, and difficulty paying bills or reporting only non-married status. Results were also generally similar for vegetable variety. More specifically, in men, the combination of low economic resources and being non-married showed a magnitude of association with both variety scores that was more than twice the individual association of social class, education or paying bills. In women, when low social class and difficulty paying bills combined with non-married, the association with lower vegetable variety was also more than double the results for social class and paying bills which only adjusted for marital status. Additionally, we further noted fruit variety was significantly lower in men but not in women, compared to reference groups, when they reported being non-married and high economic resources.

**Fig. 2:**
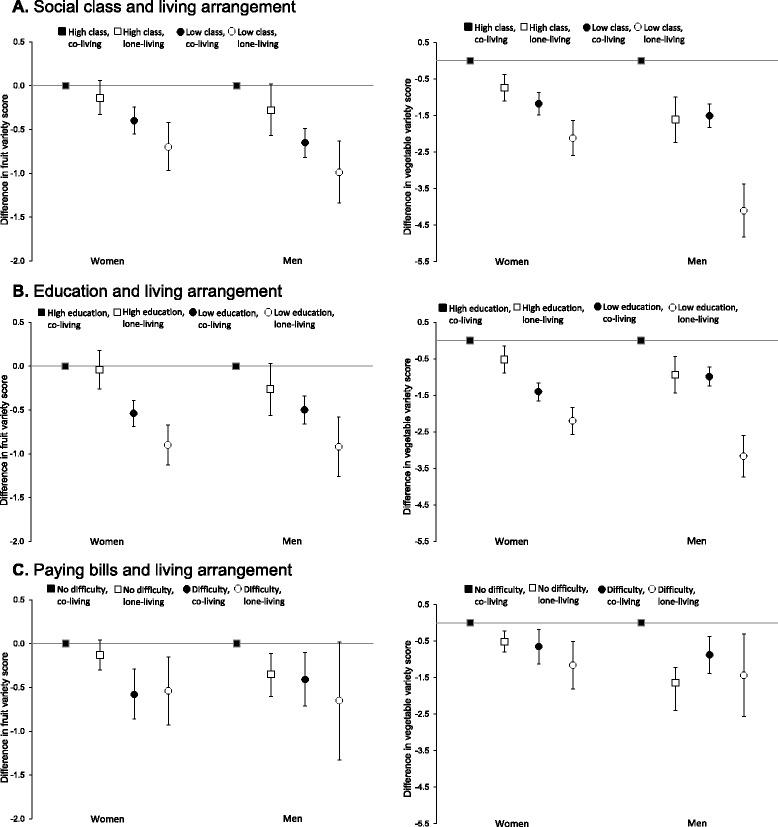
Association of social class, education and difficulty paying bills with variety of fruit or vegetable intake in older women and men by living arrangement. Left, fruit variety; Right, vegetable variety. Panel **a** is social class and living arrangement (*n* = 8663); **b**, education and living arrangement (*n* = 8810); **c**, difficulty paying bills and living arrangement (*n* = 8724)

**Fig. 3:**
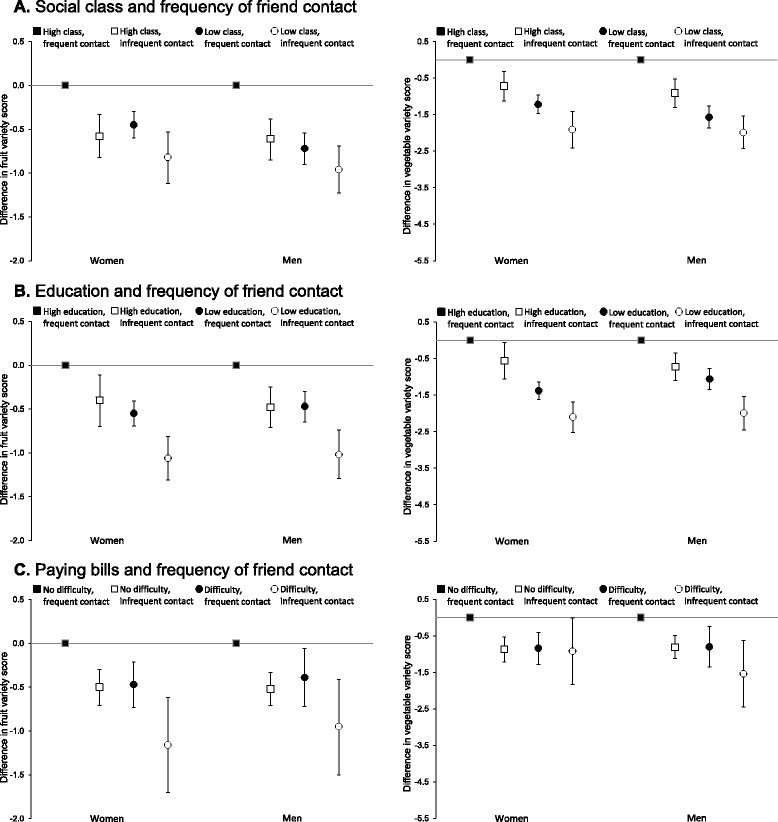
Association of social class, education and difficulty paying bills with variety of fruit or vegetable intake in older women and men by frequency of friend contact. Left, fruit variety; Right, vegetable variety. Panel **a** is social class and frequency of friend contact (*n* = 8298); **b**, education and frequency of friend contact (*n* = 8437); **c**, difficulty paying bills and frequency of friend contact (*n* = 8396)

The pattern of results was similar when different living arrangements were combined with education, social class and paying bills (Fig. 2). Again, we observed disproportionately lower variety of fruit or vegetable intakes among those reporting both low economic resources and lone-living, particularly in lone-living men reporting low education or difficulty paying bills and in lone-living women reporting difficulty paying bills. Finally, frequency of friend contact also combined with each economic resource variable to reveal notable heterogeneity in the association with fruit variety or vegetable variety for both women and men (Fig. 3). More specifically, infrequent friend contact was the only social relationship to be consistently associated with lower fruit variety in women reporting high economic resources. Thus, women and men reporting low economic resources, infrequent friend contact, or both, had lower variety of intake of fruits and vegetables compared to reference groups. Again, disproportionately lower variety scores were seen among individuals reporting both difficulty paying bills and infrequent friend contact.

Values and results for inter-relations between all six economic resources and all three social relationships are available online (see Additional file 1: Tables S2, S3 and S4).

## Discussion

This study revealed independent associations between multiple economic resources, or social relationships, and variety of FV intake in older British adults. Of greatest novelty was the demonstration that variety of fruit or vegetable intake was much lower for each measure of economic disadvantage when individuals also lacked a social relationship. As hypothesized, women and men differed in which configurations of economic resource and social relationships were most strongly associated with variety of FV consumption. We also found that, regardless of economic resources, only men consumed fewer different fruits or vegetables when non-married or lone-living.

### Relationship to previous work

Economic factors as well as social relationships are known to influence diet quality, particularly FV consumption, in older adults [6, 7, 9]. Yet, the potential linkages between economic factors and social relationships as a unique determinant of healthy eating in older adults remains an identified evidence gap [9]. This study is the first, to our knowledge, to assess inter-relations of a diverse range of economic resource measures and social relationships with respect to two markers of healthy eating in older adults.

Limited research suggests that the combination of economic and social factors has a unique impact on health [32–36]. A prospective US study showed employment status altered the influence of cohabitation on 6-year weight gain in young men [33]. In cross-sectional studies of Swedish or UK populations, social capital (including contact with friends) modified adverse effects of economic deprivation on mental health [34–36]. Overall, evidence supports the notion of a synergy of action resulting in an interlocking disadvantage of low social resources being more concentrated in economically poorer groups [32, 37, 38]. In other words, lower status persons experience a pervasive disadvantage in exposure and vulnerability to poor health from undesirable life events including marital termination [38].

The synergy effects reported for health outcomes parallel our findings that an older person’s lack of a social relationship magnified the associations between disadvantage in a given economic resource and lower variety (i.e. less healthy eating). We also found consistent associations between an absent social relationship and lower variety among men reporting economic advantage. Although some report associations of low social resources with poorer health only among people in economically deprived circumstances [34], our results concur with other work showing that poor social capital or low social cohesion were associated with poor health outcomes regardless of economic poverty [32, 35, 36].

More importantly, we examined inter-relations of multiple dimensions of economic disadvantage and poor social relationships as deemed necessary to account for highly differentiated lived experiences that influence risk factors of cardio-metabolic conditions [39]. Given the consistent associations across the broad set of indicators, we can interpret results as the constraint on older persons’ life choices about healthy eating, particularly variety of FV intake, from either a lack of capabilities in several areas [36], or as intersecting and mutually reinforcing disadvantages [14]. Several results were consistent with Intersectionality Theory as they illustrated how disadvantage in one context limited the realization of status/resources in another context such that the net effect was greater than the sum of individual disadvantages [13, 14]. In other words, social relationships constituted a resource generating economic resources and vice versa, with the absence of both creating a double burden of intersecting disadvantage with a unique impact on healthy eating. Our results indicated that disadvantageous economic and social resources should not be viewed as exclusive or separate determinants of healthy eating in older adults. Rather, their combined associations with variety of FV intakes reflected the unique constellation of highly differentiated economic and social categories that older people occupied [9], and therefore requires a novel public health approach to account for this complex reality [14].

Our results also confirmed women and men were differentially vulnerable to less healthy eating from combined economic and social disadvantages [16]. Overall, findings demonstrated that men, particularly non-married or lone-living, fared worse in the associations between dual economic-social disadvantages and healthy eating. For example, economically disadvantaged non-married men ate 1.3 fewer different vegetables daily than women counterparts; economically disadvantaged lone-living men ate 1.0 less unique vegetable daily than similarly disadvantaged women. Economic disadvantages combined with infrequent friend contact were also generally worse for men’s vegetable variety. However, in terms of fruit variety, women fared worse than men since women ate 0.5 fewer different fruits daily than men when they reported both economic disadvantage and infrequent friend contact. Results therefore supported our hypothesis that women and men differed in the specific combinations of low economic resources and absent social relationships that were most strongly associated with less healthy eating. As suggested by previous work, it is possible that the observed double burden on variety of FV intakes was worse in men because they may perceive dual economic-social disadvantages as deprivation while women may experience similar intersecting disadvantages as status quo [40].

### Methodological considerations

Recall or social desirability bias may have affected our self-reported exposures and outcomes. As we have argued previously [7, 24], perceptions of economic or social resources are worth investigating as subjective levels may predict dietary behaviours more than objective levels [6]. Moreover, the construction of set meal routines and consumption patterns may mitigate potential diet recall error and bias [7]. Nevertheless, the study design did not account for transitions in, or cumulative economic or social disadvantage, that could alter associations in opposing directions. Any misclassification of exposures from changes to participants’ economic resources or social relationships between surveys, however, would be non-differential as misclassification would be unrelated to dietary outcomes; thus results would be biased towards the null.

Residual confounding by income that was not collected in this cohort might bias observed associations to be larger than true associations. Although low income can be a barrier to FV consumption, current income is not the only resource older adults can use for food expenditures and could explain why income is inconsistently associated with diet quality [9]. Residual confounding might also occur from unexamined aspects of social relationships, including the functional aspects [41]. Finally, findings cannot be generalized to lower SES populations, or to non-white or younger groups. Nevertheless, the analytic sample showed similar characteristics to the full cohort among responders and among non-responders. There were small differences in characteristics between responders and non-responders in the analytic sample, but these were also seen in the full cohort, suggesting there were no unequal probabilities of selection and non-response rate.

This study has numerous strengths: a large sample size, gender-specific analyses, six economic factors, three social relationships and two dietary outcomes. A proxy for wealth was included among the conventional SES indicators strongly associated with diet quality in older adults [42], and older adults’ financial situation was examined using three financial hardship measures which can be experienced regardless of income or SES level [43]. It is recommended to examine multiple potentially relevant economic factors in relation to key socio-demographic characteristics, including gender, not least because people who are similar on a single factor may not be economically comparable and different types of economic exposure may have unique associations with diet quality [44, 45]. Several social relationships also have distinct associations with older adults’ healthy eating and thus separate examination is warranted [7]. But, the particular strength of this work was in considering the inter-relations of diverse economic resources and social relationships from a gender perspective. In doing so, this study begins to capture the complex reality of an older individual’s heterogeneous life circumstances wherein multiple social roles and diverse economic resources intersect to produce unique configurations that are specific to women and men and have distinct influences on healthy eating [9, 13, 17].

## Conclusion

This study confirms that the combination of all forms of economic and social disadvantage showed much lower variety of fruit or vegetable intakes than when either disadvantage was considered alone. It also contributes new knowledge on the gender-specific effects, as men fared worse overall than women in the double burden of intersecting disadvantages on healthy eating. Thus, public health efforts to increase variety of FV in older adults will benefit from simultaneously addressing their financial situation and social connectivity. Results also highlight the importance of considering gender when assessing which combination of economic and social disadvantages might be targeted in the promotion of healthy eating.

## Additional file

Additional file 1:
**Tables S1, S2, S3 and S4.**
