# Supplementary material for: Gender and the double burden of economic and social disadvantages on healthy eating: cross-sectional study of older adults in the EPIC-Norfolk cohort
Source: BMC Public Health. 2015 Jul 22;15:692. doi: 10.1186/s12889-015-1895-y (PMC4511519; doi:10.1186/s12889-015-1895-y)
Supplement: Additional file 1: — Tables S1, S2, S3 and S4. [file 12889_2015_1895_MOESM1_ESM.docx]

**TABLE S1** Associations between economic resources and variety of fruit or vegetable intakes in older adults in the EPIC-Norfolk study

|  | **Fruit Variety** | | | |  | **Vegetable Variety** | | | |
| --- | --- | --- | --- | --- | --- | --- | --- | --- | --- |
|  | **Women** | | **Men** | |  | **Women** | | **Men** | |
|  | ***Model 1*** | ***Model 2*** | ***Model 1*** | ***Model 2*** |  | ***Model 1*** | ***Model 2*** | ***Model 1*** | ***Model 2*** |
| **Home-ownership** | | | | | | | | | |
| Owner-occupier | reference | reference | reference | reference |  | reference | reference | reference | reference |
| Renter-occupier | -0.71  (-0.94, -0.47) | -0.58  (-0.88, -0.29) | -0.83  (-1.12, -0.54) | -0.77  (-1.14, -0.40) |  | -1.48  (-1.88, -1.09) | -1.47  (-1.97, -0.98) | -1.72  (-2.20, -1.23) | -1.53  (-2.15, -0.90) |
|  | *** | *** | ***** | *** |  | *** | *** | *** | *** |
| **Money for needs** | | | | | | | | | |
| More than enough | reference | reference | reference | reference |  | reference | reference | reference | reference |
| Just or less than enough | -0.33  (-0.50, -0.16) | -0.30  (-0.50, -0.10) | -0.46  (-0.65, -0.28) | -0.35  (-0.58, -0.13) |  | -0.52  (-0.81, -0.24) | -0.52  (-0.86, -0.18) | -0.81  (-1.12, -0.50) | -0.92  (-1.29, -0.54) |
|  | ***** | **** | ***** | **** |  | ***** | **** | ***** | ***** |
| **Frequency of insufficient money for food/clothing** | | | | | | | | | |
| Infrequent | reference | reference | reference | reference |  | reference | reference | reference | reference |
| Frequent | -0.45  (-0.62, -0.27) | -0.40**^ⱡ^**  (-0.61, -0.19) | -0.33  (-0.54, -0.13) | -0.09**^ⱡ^**  (-0.35, 0.17) |  | -0.52  (-0.81, -0.23) | -0.32  (-0.68, 0.04) | -0.69  (-1.04, -0.34) | -0.69  (-1.13, -0.26) |
|  | ***** | ***** | **** |  |  | ***** |  | ***** | **** |
| Gender-specific beta coefficients (CI95) obtained by linear regression models using an interaction term and adjusting for age and energy intake (Model 1), and then for all three social relationships (Model 2). Numbers were: home-ownership (Model 1: 8,701; Model 2: 5,531); money for needs (Model 1: 8,747; Model 2: 5,572); frequency of insufficient money for food/clothing (Model 1: 8,753; Model 2: 5,579). *p<0.05, **p<0.01, ***p<0.001; **^ⱡ^**Significant gender difference (p-interaction<0.10). | | | | | | | | | |

**TABLE S2** Association of inter-relations between economic resources and marital status with variety of fruit or vegetable intakes in older women and men in the EPIC-Norfolk study

|  | **Fruit variety** | | | | |
| --- | --- | --- | --- | --- | --- |
|  | **Women** | |  | **Men** | |
|  | ***Marital status*** | |  | ***Marital status*** | |
|  | ***Married*** | ***Non-married*** |  | ***Married*** | ***Non-married*** |
| **Social class (n=6,151)** | | |  |  |  |
| High | reference | 0.02**^ⱡ^**  (-0.20, 0.24) |  | reference | -0.50**^ⱡ^**  (-0.87, -0.13)** |
| Low | -0.37**^ⱡ^**  (-0.55, -0.18)*** | -0.76**^ⱡ^**  (-1.05, -0.46)*** |  | -0.62**^ⱡ^**  (-0.81, -0.43)*** | -1.34**^ⱡ^**  (-1.78, -0.91)*** |
| **Education (n=6,252)** | | |  |  |  |
| High | reference | 0.03**^ⱡ^**  (-0.21, 0.28) |  | reference | -0.37**^ⱡ^**  (-0.74, -0.01)* |
| Low | -0.54  (-0.72, -0.37)*** | -0.80**^ⱡ^**  (-1.06, -0.55)*** |  | -0.38  (-0.57, -0.19)*** | -1.30**^ⱡ^**  (-1.72, -0.87)*** |
| **Home-ownership (n=5,810)** | | |  |  |  |
| Owner-occupier | reference | -0.00**^ⱡ^**  (-0.20, 0.19) |  | reference | -0.40**^ⱡ^**  (-0.73, -0.08)* |
| Renter-occupier | -0.58  (-0.92, -0.18)** | -0.75  (-1.15, -0.35)*** |  | -0.82  (-1.24, -0.40)*** | -1.07  (-1.69, -0.44)*** |
| **Money for needs (n=5,830)** | | |  |  |  |
| More than enough | reference | 0.11**^ⱡ^**  (-0.34, 0.57) |  | reference | -1.14**^ⱡ^**  (-1.79, -0.48)*** |
| Just or less than enough | -0.28  (-0.50, -0.05)* | -0.37**^ⱡ^**  (-0.63, -0.10)** |  | -0.50  (-0.73, -0.26)*** | -0.85**^ⱡ^**  (-1.22, -0.49)*** |
| **Frequency of insufficient money for food/clothing (n=5,836)** | | |  |  |  |
| Infrequent | reference | 0.10**^ⱡ^**  (-0.10, 0.31) |  | reference | -0.47**^ⱡ^**  (-0.79,-0.14)** |
| Frequent | -0.25  (-0.51, 0.01) | -0.71  (-1.04, -0.39)*** |  | -0.09  (-0.36, 0.18) | -0.77  (-1.36, -0.17)* |
| **Paying bills (n=5,839)** | | |  |  |  |
| No difficulty | reference | 0.02**^ⱡ^**  (-0.18, 0.21) |  | reference | -0.51**^ⱡ^**  (-0.82, -0.20)*** |
| Difficulty | -0.46  (-0.85, -0.08)* | -0.67  (-1.06, -0.27)*** |  | -0.16  (-0.53, 0.21) | -0.66  (-1.44, 0.11) |
|  | **Vegetable variety** | | | | |
|  | **Women** | |  | **Men** | |
|  | ***Marital status*** | |  | ***Marital status*** | |
|  | ***Married*** | ***Non-married*** |  | ***Married*** | ***Non-married*** |
| **Social class (n=6,151)** | | |  |  |  |
| High | reference | -0.74**^ⱡ^**  (-1.10, -0.37)*** |  | reference | -1.61**^ⱡ^**  (-2.24, -0.99)*** |
| Low | -1.18  (-1.49, -0.87)*** | -2.12**^ⱡ^**  (-2.60, -1.63)*** |  | -1.51  (-1.83, -1.19)*** | -4.11**^ⱡ^**  (-4.83, -3.38)*** |
| **Education (n=6,252)** | | |  |  |  |
| High | reference | -0.70**^ⱡ^**  (-1.11, -0.29)*** |  | reference | -1.34**^ⱡ^**  (-1.95, -0.72)*** |
| Low | -1.50**^ⱡ^**  (-1.80, -1.20)*** | -2.48**^ⱡ^**  (-2.90, -2.06)*** |  | -0.94**^ⱡ^**  (-1.26, -0.62)*** | -3.89**^ⱡ^**  (-4.60, -3.18)*** |
| **Home-ownership (n=5,810)** | | |  |  |  |
| Owner-occupier | reference | -0.57**^ⱡ^**  (-0.90, -0.24)*** |  | reference | -1.86**^ⱡ^**  (-2.41, -1.32)*** |
| Renter-occupier | -1.68  (-2.34, -1.02)*** | -2.07  (-2.74, -1.40)*** |  | -1.60  (-2.31, -0.90)*** | -2.74  (-3.80, -1.69)*** |
| **Money for needs (n=5,830)** | | |  |  |  |
| More than enough | reference | -1.21**^ⱡ^**  (-1.97, -0.45)** |  | reference | -3.40**^ⱡ^**  (-4.51, -2.30)*** |
| Just or less than enough | -0.71**^ⱡ^**  (-1.09, -0.33)*** | -1.34**^ⱡ^**  (-1.78, -0.90)*** |  | -1.17**^ⱡ^**  (-1.57, -0.78)*** | -2.77**^ⱡ^**  (-3.39, -2.15)*** |
| **Frequency of insufficient money for food/clothing (n=5,836)** | | |  |  |  |
| Infrequent | reference | -1.85**^ⱡ^**  (-2.40, -1.30)*** |  | reference | -0.75**^ⱡ^**  (-1.10, -0.40)*** |
| Frequent | -0.63  (-1.08, -0.17)** | -2.83**^ⱡ^**  (-3.83, -1.84)*** |  | -0.48  (-0.92, -0.04)* | -1.00**^ⱡ^**  (-1.55, -0.46)*** |
| **Paying bills (n=5,839)** | | |  |  |  |
| No difficulty | reference | -0.65**^ⱡ^**  (-0.98, -0.32)*** |  | reference | -2.08**^ⱡ^**  (-2.60, -1.56)*** |
| Difficulty | -0.25  (-0.89, 0.40) | -1.32  (-1.99, -0.66)*** |  | -0.75  (-1.38, -0.12)* | -1.56  (-2.87, -0.26)* |

Gender-specific beta coefficients (CI95) obtained by linear regression models using an interaction term and adjusting for age and total energy intake (kcal/d). *p<0.05, **p<0.01, ***p<0.001; **^ⱡ^**Significant gender difference in a given exposure category (p-interaction<0.10).

**TABLE S3** Association of inter-relations between economic resources and living arrangement with variety of fruit or vegetable intakes in older women and men in the EPIC-Norfolk study

|  | **Fruit variety** | | | | |
| --- | --- | --- | --- | --- | --- |
|  | **Women** | |  | **Men** | |
|  | ***Living arrangement*** | |  | ***Living arrangement*** | |
|  | ***Co-living*** | ***Lone-living*** |  | ***Co-living*** | ***Lone-living*** |
| **Social class (n=8,663)** | | |  |  |  |
| High | reference | -0.14  (-0.33, 0.06) |  | reference | -0.28  (-0.57, 0.02) |
| Low | -0.40**^ⱡ^**  (-0.55, -0.24)*** | -0.70  (-0.97, -0.42)*** |  | -0.65**^ⱡ^**  (-0.57, -0.49)*** | -0.99  (-1.34, -0.63)*** |
| **High education (n=8,810)** | | |  |  |  |
| High | reference | -0.04  (-0.26, 0.18) |  | reference | -0.26  (-0.56, 0.03) |
| Low | -0.54  (-0.69, -0.39)*** | -0.90  (-1.13, -0.67)*** |  | -0.50  (-0.66, -0.34)*** | -0.92  (-1.26, -0.58)*** |
| **Home-ownership (n=8,681)** | | |  |  |  |
| Owner-occupier | reference | -0.10  (-0.27, 0.08) |  | reference | -0.28  (-0.54, -0.03)* |
| Renter-occupier | -0.66  (-0.96, -0.35)*** | -0.83  (-1.19, -0.47)*** |  | -0.85  (-1.20, -0.50)*** | -0.85  (-1.36, -0.35)*** |
| **Money for needs (n=8,709)** | | |  |  |  |
| More than enough | reference | -0.10  (-0.49, 0.30) |  | reference | -0.55  (-1.07, -0.03)* |
| Just or less than enough | -0.31  (-0.50, -0.12)*** | -0.47  (-0.70, -0.24)*** |  | -0.49  (-0.68, -0.29)*** | -0.80  (-1.10, -0.50)*** |
| **Frequency of insufficient money for food/clothing (n=8,715)** | | |  |  |  |
| Infrequent | Reference | -0.05  (-0.23, 0.14) |  | reference | -0.36  (-0.62, -0.10)** |
| Frequent | -0.35  (-0.55, -0.14)*** | -0.69  (-0.99, -0.39)*** |  | -0.35  (-0.57, -0.12)** | -0.56  (-1.03, -0.09)* |
| **Paying bills (n=8,724)** | | |  |  |  |
| No Difficulty | reference | -0.13  (-0.30, 0.04) |  | reference | -0.35  (-0.60, -0.11)** |
| Difficulty | -0.58  (-0.86, -0.29)*** | -0.54  (-0.93, -0.15)** |  | -0.41  (-0.71, -0.10)** | -0.65  (-1.33, 0.02) |
|  | **Vegetable variety** | | | | |
|  | **Women** | |  | **Men** | |
|  | ***Living arrangement*** | |  | ***Living arrangement*** | |
|  | ***Co-living*** | ***Lone-living*** |  | ***Co-living*** | ***Lone-living*** |
| **Social class (n=8,663)** | | |  |  |  |
| High | Reference | -0.63**^ⱡ^**  (-0.95, -0.30)*** |  | reference | -1.28**^ⱡ^**  (-1.77, -0.78)*** |
| Low | -1.21  (-1.47, -0.95)*** | -1.88**^ⱡ^**  (-2.33, -1.43)*** |  | -1.48  (-1.75, -1.21)*** | -3.05**^ⱡ^**  (-3.64, -2.46)*** |
| **High education (n=8,810)** | | |  |  |  |
| High | Reference | -0.52  (-0.89, -0.15)** |  | reference | -0.94  (-1.43, -0.44)*** |
| Low | -1.40**^ⱡ^**  (-1.65, -1.16)*** | -2.20**^ⱡ^**  (-2.57, -1.83)*** |  | -0.99**^ⱡ^**  (-1.25, -0.72)*** | -3.17**^ⱡ^**  (-3.73, -2.60)*** |
| **Home-ownership (n=8,681)** | | |  |  |  |
| Owner-occupier | Reference | -0.57**^ⱡ^**  (-0.85, -0.28)*** |  | reference | -1.48**^ⱡ^**  (-1.90, -1.06)*** |
| Renter-occupier | -1.81  (-2.32, -1.30)*** | -1.35  (-1.95, -0.75)*** |  | -1.77  (-2.35, -1.19)*** | -2.10  (-2.95, -1.25)*** |
| **Money for needs (n=8,709)** | | |  |  |  |
| More than enough | Reference | -1.08**^ⱡ^**  (-1.74, -0.41)*** |  | reference | -2.03**^ⱡ^**  (-2.90, -1.17)*** |
| Just or less than enough | -0.61  (-0.93, -0.30)*** | -1.08**^ⱡ^**  (-1.46, -0.69)*** |  | -0.88  (-1.20, -0.55)*** | -2.32**^ⱡ^**  (-2.82, -1.83)*** |
| **Frequency of insufficient money for food/clothing (n=8,715)** | | |  |  |  |
| Infrequent | Reference | -0.59  (-0.89, -0.29)*** |  | reference | -1.49  (-1.92, -1.06)*** |
| Frequent | -0.54  (-0.89, -0.20)** | -0.82  (-1.32, -0.33)*** |  | -0.57  (-0.95, -0.19)** | -2.10  (-2.88, -1.32)*** |
| **Paying bills (n=8,724)** | | |  |  |  |
| No Difficulty | Reference | -0.52**^ⱡ^**  (-0.80, -0.23)*** |  | reference | -1.64**^ⱡ^**  (-2.40, -1.23)*** |
| Difficulty | -0.65  (-1.13, -0.18)** | -1.16  (-1.81, -0.51)*** |  | -0.88  (-1.39, -0.37)*** | -1.44  (-2.56, -0.31)* |

Gender-specific beta coefficients (CI95) obtained by linear regression models using an interaction term and adjusting for age and total energy intake (kcal/d). *p<0.05, **p<0.01, ***p<0.001; **^ⱡ^**Significant gender difference in a given exposure category (p-interaction<0.10).

**TABLE S4** Association of inter-relations between economic resources and friend contact with variety of fruit or vegetable intakes in older women and men in the EPIC-Norfolk study

|  | **Fruit variety** | | | | |
| --- | --- | --- | --- | --- | --- |
|  | **Women** | |  | **Men** | |
|  | ***Friend contact*** | |  | ***Friend contact*** | |
|  | ***Frequent*** | ***Infrequent*** |  | ***Frequent*** | ***Infrequent*** |
| **Social class (n=8,298)** | | |  |  |  |
| High | reference | -0.58  (-0.82, -0.33)*** |  | reference | -0.61  (-0.85, -0.38)*** |
| Low | -0.45**^ⱡ^**  (-0.60, -0.30)*** | -0.82  (-1.12, -0.53)*** |  | -0.72**^ⱡ^**  (-0.90, -0.54)*** | -0.96  (-1.23, -0.69)*** |
| **Education (n=8,437)** | | |  |  |  |
| High | reference | -0.40  (-0.70, -0.11)** |  | reference | -0.48  (-0.71, -0.25)*** |
| Low | -0.55  (-0.69, -0.41)*** | -1.06  (-1.31, -0.81)*** |  | -0.47  (-0.65, -0.30)*** | -1.02  (-1.29, -0.74)*** |
| **Home-ownership (n=8,300)** | | |  |  |  |
| Owner-occupier | reference | -0.46  (-0.66, -0.25)*** |  | reference | -0.55  (-0.74, -0.37)*** |
| Renter-occupier | -0.54  (-0.80, -0.27)*** | -1.59  (-2.16, -1.01)*** |  | -0.85  (-1.18, -0.51)*** | -1.11  (-1.75, -0.47)*** |
| **Money for needs (n=8,381)** | | |  |  |  |
| More than enough | reference | -0.80  (-1.34, 0.27)** |  | reference | -0.60  (-1.03, -0.18)** |
| Just or less than enough | -0.33  (-0.51, -0.15)*** | -0.80  (-1.05, -0.55)*** |  | -0.45  (-0.65, -0.24)*** | -0.94  (-1.19, -0.68)*** |
| **Frequency of insufficient money for food/clothing (n=8,388)** | | |  |  |  |
| Infrequent | reference | -0.46  (-0.67, -0.24)*** |  | reference | -0.54  (-0.73, -0.34)*** |
| Frequent | -0.35  (-0.55, -0.16)** | -1.16  (-1.55, -0.76)*** |  | -0.33  (-0.57, -0.09)*** | -0.78  (-1.20, -0.37)*** |
| **Paying bills (n=8,396)** | | |  |  |  |
| No difficulty | reference | -0.50  (-0.71, -0.30)*** |  | reference | -0.52  (-0.71, -0.33)*** |
| Difficulty | -0.47  (-0.73, -0.21)*** | -1.16  (-1.70, -0.62)*** |  | -0.39  (-0.72, -0.06)* | -0.95  (-1.50, -0.41)*** |
|  | **Vegetable variety** | | | | |
|  | **Women** | |  | **Men** | |
|  | ***Friend contact*** | |  | ***Friend contact*** | |
|  | ***Frequent*** | ***Infrequent*** |  | ***Frequent*** | ***Infrequent*** |
| **Social class (n=8,298)** | | |  |  |  |
| High | reference | -0.72  (-1.13, -0.31)*** |  | reference | -0.91  (-1.30, -0.52)*** |
| Low | -1.22**^ⱡ^**  (-1.47, -0.96)*** | -1.91  (-2.41, -1.42)*** |  | -1.57**^ⱡ^**  (-1.87, -1.27)*** | -1.99  (-2.43, -1.54)*** |
| **Education (n=8,437)** | | |  |  |  |
| High | reference | -0.56  (-1.05, -0.06)*** |  | reference | -0.72  (-1.10, -0.34)*** |
| Low | -1.38**^ⱡ^**  (-1.62, -1.14)*** | -2.10  (-2.52, -1.69)*** |  | -1.06**^ⱡ^**  (-1.35, -0.77)*** | -1.99  (-2.45, -1.53)*** |
| **Home-ownership (n=8,300)** | | |  |  |  |
| Owner-occupier | reference | -0.64  (-0.98, -0.30)*** |  | reference | -0.84  (-1.15, -0.53)*** |
| Renter-occupier | -1.25  (-1.69, -0.80)*** | -2.76  (-3.73, -1.80)*** |  | -1.75  (-2.32, -1.19)*** | -2.36  (-3.42, -1.29)*** |
| **Enough money for needs (n=8,381)** | | |  |  |  |
| More than enough | reference | -0.99  (-1.89, -0.10)* |  | reference | -0.61  (-1.32, -0.10)* |
| Just or less than enough | -0.48  (-0.78, -0.18)** | -1.22  (-1.63, -0.80)*** |  | -0.74  (-1.09, -0.40)*** | -2.32  (-2.82, -1.83)*** |
| **Frequency of insufficient money to afford food/clothing (n=8,388)** | | |  |  |  |
| Infrequent | reference | -0.79  (-1.15, -0.43)*** |  | reference | -0.80  (-1.13, -0.47)*** |
| Frequent | -0.45  (-0.77, -0.12)** | -1.27  (-1.94, -0.60)*** |  | -0.70  (-1.11, -0.30)*** | -1.54  (-2.23, -0.85)*** |
| **Paying bills (n=8,396)** | | |  |  |  |
| No difficulty | reference | -0.87  (-1.21, -0.53)*** |  | reference | -0.81  (-1.12, -0.49)*** |
| Difficulty | -0.84  (-1.28, -0.41)*** | -0.92  (-1.83, -0.01)* |  | -0.80  (-1.35, -0.24)** | -1.54  (-2.45, -0.63)*** |

Gender-specific beta coefficients (CI95) obtained by linear regression models using an interaction term and adjusting for age and total energy intake (kcal/d). *p<0.05, **p<0.01, ***p<0.001; **^ⱡ^**Significant gender difference in a given exposure category (p-interaction<0.10).
